# Hypercoagulability in Tuberculosis: Pathophysiological Mechanisms, Associated Risks, and Advances in Management—A Narrative Review

**DOI:** 10.3390/jcm14030762

**Published:** 2025-01-24

**Authors:** Denisa Maria Mitroi, Mara Amalia Balteanu, Ramona Cioboata, Silviu Gabriel Vlasceanu, Ovidiu Mircea Zlatian, Oana Maria Catana, Adina Andreea Mirea, Gabriel Florin Razvan Mogos, Ionela Rotaru, Viorel Biciusca

**Affiliations:** 1 Doctoral School, University of Medicine and Pharmacy, 200349 Craiova, Romania; denisa_maria2@yahoo.com (D.M.M.); oana_cattana@yahoo.com (O.M.C.); 2 Department of Pulmonology, Faculty of Medicine, Titu Maiorescu University, 031593 Bucharest, Romania; mara.balteanu@prof.utm.ro; 3 Pneumology Department, University of Medicine and Pharmacy, 200349 Craiova, Romania; biciuscaviorel@gmail.com; 4 Department of Physiology, “Carol Davila” University of Medicine and Pharmacy, 050474 Bucharest, Romania; 5 Microbiology Department, University of Medicine and Pharmacy of Craiova, 200349 Craiova, Romania; ovidiu.zlatian@umfcv.ro; 6 Department of Oral-Dental Prevention, University of Medicine and Pharmacy, 200349 Craiova, Romania; adinaturcu14@yahoo.com; 7 Department of Surgery, University of Medicine and Pharmacy of Craiova, 200349 Craiova, Romania; gabrielmogos@yahoo.com; 8 Department of Hematology, Faculty of Medicine, University of Medicine and Pharmacy of Craiova, 200349 Craiova, Romania; ionela.rotaru@umfcv.ro

**Keywords:** tuberculosis, hypercoagulability, disease management

## Abstract

Tuberculosis (TB) induces a hypercoagulable state characterized by systemic inflammation, endothelial dysfunction, and alterations in the coagulation and fibrinolytic pathways. This review explores the pathophysiological mechanisms underlying hypercoagulability in TB, including increased pro-inflammatory cytokine release, endothelial damage, platelet activation, and reduced anticoagulant and fibrinolytic activity. These factors contribute to an elevated risk of venous thromboembolism (VTE), including deep vein thrombosis (DVT) and pulmonary embolism (PE), which complicate TB prognosis and treatment. The potential role of adjunctive anti-inflammatory therapies, such as vitamin D, NSAIDs, corticosteroids, and anti-platelet agents, is highlighted as a strategy to mitigate systemic inflammation and reduce thrombotic risks in patients with TB. The challenges of anticoagulation therapy, particularly in managing the interactions between anti-TB medications and traditional anticoagulants, are discussed, along with the potential of novel oral anticoagulants (NOAs) as alternatives. We also address therapy of hypercoagulability in TB within resource-limited settings which requires low-cost diagnostics, accessible anticoagulation options, adjunctive therapies, and preventive strategies integrated into existing healthcare systems. Effective risk stratification and individualized management strategies are vital for reducing the morbidity and mortality associated with thrombotic complications in TB.

## 1. Introduction

Tuberculosis (TB), caused by *Mycobacterium tuberculosis* (Mtb), is an airborne-transmitted disease that primarily affects the respiratory system. It remains a leading infectious cause of morbidity and mortality worldwide, with significant public health implications, particularly in low- and middle-income countries, where resources for prevention, early diagnosis, and treatment are constrained. In 2022, approximately 10.6 million individuals (95% uncertainty interval: 9.9–11.4 million) were infected, with 7.5 million cases detected globally and around 1.30 million fatalities (95% uncertainty interval: 1.18–1.43 million) [1,2].

Although TB primarily affects the lungs, it can spread hematogenously or through lymphatic dissemination, leading to extrapulmonary TB, which can affect almost any organ: the lymph nodes, pleura, bones, central nervous system, and the genitourinary system [3]. This wide spectrum of clinical presentation makes TB a complex disease with multiple systemic manifestations beyond its classic respiratory symptoms [4]. In addition to its respiratory impact, TB can influence systemic physiology, including coagulation pathways, contributing to a hypercoagulable state [5].

Thrombosis, particularly venous thromboembolism (VTE), is a notable concern in patients with active tuberculosis (TB). Epidemiological data highlight an elevated risk of VTE in individuals with TB, including deep vein thrombosis (DVT) and pulmonary embolism (PE). A meta-analysis by Danwang et al. [6] encompassing nine studies with a total of 16,190 TB patients, estimated the prevalence of VTE in active TB cases to be approximately 3.5%, with pulmonary embolism accounting for 5.8% and deep vein thrombosis for 1.3%. Furthermore, patients with active TB were found to have a significantly increased risk of VTE (odds ratio (OR 2.90)), DVT (OR 1.56), and PE (OR 3.58) compared to those without TB. Supporting these findings, a retrospective observational cohort study by Lau et al. [7] involving 240 hospitalized patients with pulmonary TB revealed that 4.2% were diagnosed with VTE.

The association between pulmonary embolism (PE) and tuberculosis (TB) has been recognized in the literature since the 1950s. Moran’s analysis of autopsy reports in patients with active tuberculosis revealed that 24.3% had pulmonary embolism [8]. Similarly, a report by Vaideeswar and Deshpande [9] evaluating 30 cases of aortic thrombosis identified six patients with active tuberculosis, further emphasizing the link between TB and thrombotic complications. Additionally, Robson et al. [10] reported that 2 out of 35 patients had deep vein thrombosis (DVT) at admission, while the remaining patients developed DVT after one week of anti-TB treatment.

In tuberculosis, various inflammatory cells, cytokines, and immune effectors play a significant role in the formation of granulomatous lesions characteristic of the disease [11]. In response to the infection, immune cells produce pro-inflammatory cytokines such as interleukin (IL)-1, IL-6, and tumor necrosis factor (TNF)-α, which disrupt homeostasis [12]. These disruptions include increased pro-coagulant activity (evident through changes in prothrombin time (PT), activated partial thromboplastin time (aPTT), fibrinogen, and D-dimer levels), decreased anticoagulant factors (including antithrombin III, protein S, and protein C), and suppressed fibrinolysis, ultimately resulting in a hypercoagulable state [13,14]. This phenomenon can increase the risk of venous thromboembolism (VTE), which includes conditions such as deep vein thrombosis (DVT) and pulmonary embolism (PE), complicating TB prognosis and treatment [7,15].

Understanding these pathological mechanisms is critical for the early identification and management of thrombotic complications in TB patients, which can significantly affect their prognosis. This review examines the mechanisms of hypercoagulability in tuberculosis, its clinical implications, and therapeutic strategies, including the challenges of anticoagulation, the potential of novel oral anticoagulants, and alternative options like low-molecular-weight heparin.

## 2. Pathophysiological Mechanisms of Hypercoagulability in TB

The hemostatic process involves four phases [5]: endothelial injury and formation of the platelet plug, propagation of the clotting process by the coagulation cascade, termination of clotting by antithrombotic control mechanisms, removal of the clot by fibrinolysis. Any impairment of one of these phases results in hemorrhagic or thrombotic complications.

**Figure 1:**
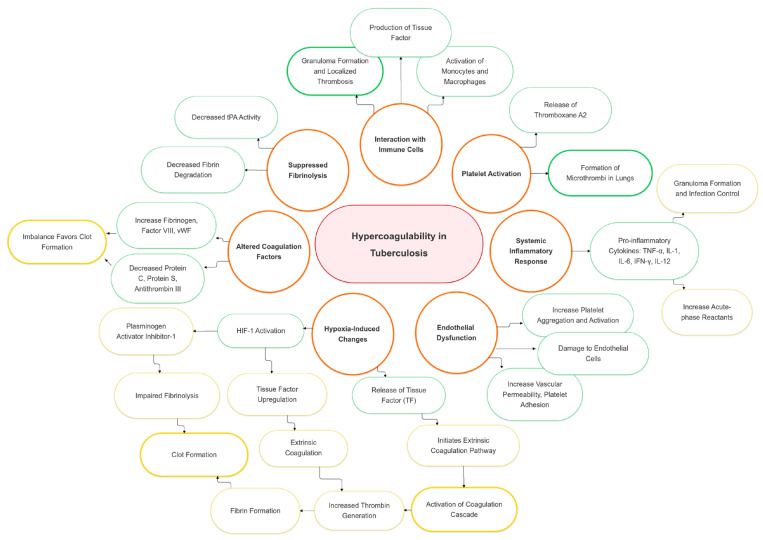
Pathophysiological mechanisms and risk factors of hypercoagulability in tuberculosis. HIF-1: hypoxia-inducible factor 1; human IL-1: interleukin 1; IL-6: interleukin 2; IL-12: interleukin 6; IFN- γ: interferon-gamma; TF: tissue factor; TNF-α: tumor necrosis factor α; tPA: tissue plasminogen activator.

The hypercoagulable state in tuberculosis arises from multiple mechanisms, including protein overexpression [5], enhanced coagulation activation (elevated thrombin–antithrombin complexes, D-dimer, fibrinogen) [16], impaired anticoagulant pathways (reduced antithrombin, protein C, free protein S) [17], inflammation driven by cytokines and platelets [18], tissue factor upregulation by *Mycobacterium tuberculosis* [19], and an acute-phase response with elevated fibrinogen, impaired fibrinolysis [16], and thrombocytosis [10] (Figure 1) [5,17].

### 2.1. Systemic Inflammatory Response

Tuberculosis triggers a robust immune response that involves the release of key pro-inflammatory cytokines, including tumor necrosis factor-alpha, interleukin-1, interleukin-6 (IL-6), interferon-gamma (IFN-γ), and interleukin-12 (IL-12). These cytokines play distinct but interrelated roles in the body’s attempt to control the infection. TNF-α is critical for granuloma formation, helping to contain *Mycobacterium tuberculosis* and limit its spread [12]. IL-1 promotes fever and inflammation, while IL-6 drives the acute-phase response, which includes the production of acute-phase reactants such as fibrinogen by the liver. IFN-γ is essential for activating macrophages, enhancing their bactericidal capabilities, and IL-12 supports the differentiation of T-helper 1 (Th1) cells that are key to a robust immune defense against TB [20].

This inflammatory response to TB, while essential for controlling the infection, also has prothrombotic consequences [5]. Elevated levels of IL-6 are associated with increased fibrinogen production, which may contribute to higher blood viscosity and support fibrin formation, potentially promoting a hypercoagulable state [21,22]. TNF-α plays an important role in phagocyte activation, but, at the same time, represents a mediator of tissue damage, followed by tissue factor release and cascade coagulation activation [20]. Additionally, IL-1 was shown to activate the coagulation cascade, which could exacerbate the risk of clot formation [21,23]. Consequently, the systemic inflammation linked to TB appears to play a role in creating a prothrombotic state, potentially complicating disease progression and increasing health risks [24,25].

The interplay between inflammation and coagulation in TB exemplifies the complex relationship between the immune system’s attempts to fight infection and its unintended consequences. While the immune response is essential for containing and ultimately eradicating *Mycobacterium tuberculosis*, the associated inflammatory processes can activate pathways that predispose individuals to thrombosis, complicating the clinical management of TB.

### 2.2. Endothelial Dysfunction

Endothelial dysfunction is a key factor in the development and progression of tuberculosis, significantly contributing to the hypercoagulable state associated with the disease. This dysfunction is characterized by several changes in the vascular endothelium, including alterations in key mediators such as vascular endothelial growth factor (VEGF), nitric oxide (NO), endothelin-1 (E-1), and von Willebrand factor antigen (vWF) [26]. The inflammation caused by TB leads to damage of endothelial cells lining the blood vessels, impairing their natural anticoagulant properties Damaged endothelial cells release von Willebrand factor, a protein that plays a crucial role in platelet adhesion to subendothelium and serves as a carrier for factor VIII [27]. Under normal circumstances, ADAMTS13 (a metalloproteinase) regulates von Willebrand factor levels by proteolytical degradation of the multimer. In TB patients, the ADMTS13 concentration is significantly lower and, consequently, the VW factor lever is higher, impairing the platelets adhesion [5].

The activation and injury of the endothelium significantly contribute to thrombosis in TB. The release of tissue factor activates the extrinsic coagulation pathway, leading to the production of thrombin and the subsequent formation of fibrin, promoting clot formation [28]. Additionally, the endothelium’s impaired ability to maintain vascular homeostasis results in increased vascular permeability, platelet adhesion, and aggregation, further contributing to the development of thrombosis and complicating the disease’s clinical course [29].

### 2.3. Platelet Activation

Tuberculosis-associated inflammation significantly enhances platelet activation and aggregation, contributing to a hypercoagulable state. Inflammatory cytokines, such as IL-6, increase platelet activation and promote aggregation, leading to the formation of microthrombi [21,22]. Activated platelets release pro-coagulant factors like thromboxane A2, aiding in vasoconstriction and platelet aggregation [30].

Also, in TB patients, changes in platelet granule structure have been described. Platelets in patients with acute TB contain pro-inflammatory mediators such as tumor necrosis factor alpha (TNFα) and interleukin-1 beta (IL-1β), different from platelets in patients with chronic TB which contain adenosine diphosphate (ADP), adenosine triphosphate (ATP), serotonin, and ionized calcium which is involved in the coagulation cascade [17].

Patients with active TB often exhibit elevated platelet counts correlated with disease severity and the hypercoagulable state, but these counts may decrease as the disease progresses, potentially impairing normal platelet function [31]. Thrombocytosis exacerbates the risk of thrombus formation. High platelet counts are associated with increased levels of platelet factor-4 (PF4) and other mediators that promote platelet activation [32]. Platelets accumulate in lung lesions and granulomas in TB patients, where they interact with other immune cells. These interactions can inhibit T-cell responses and *Mycobacterium tuberculosis* replication in macrophages, suggesting a dual role for platelets in both promoting and controlling infection [33].

Markers of platelet activation, such as CD62P (P-selectin) and PF4, are elevated in TB patients and correlate with the extent of pulmonary lesions [34]. Following successful treatment, these markers return to baseline levels, indicating their potential use in monitoring disease progression and treatment efficacy. Given their role in TB immunopathology, platelets are being investigated as potential targets for novel host-directed therapies. Anti-platelet agents may help limit tissue damage and improve treatment outcomes by modulating platelets’ activity and interactions with other immune cells [35].

### 2.4. Altered Coagulation Factors

Tuberculosis is associated with significant alterations in coagulation factors, contributing to a hypercoagulable state that increases the risk of thrombotic complications [36]. The complex interplay between inflammation, endothelial dysfunction, and changes in coagulation parameters disrupts normal hemostasis and promotes clot formation. Several key findings highlight the coagulation changes observed in TB patients [19].

Prothrombin time and activated partial thromboplastin time are significantly elevated in TB patients compared to healthy controls, indicating a disruption in the normal coagulation process [37]. Elevated D-dimer levels are indicative of increased fibrin degradation and are commonly associated with a hypercoagulable state, which can lead to various thrombotic complications. In a study by Alhassan and Gaufri (2017), elevated D-dimer levels were observed in Sudanese patients with pulmonary tuberculosis, highlighting a significant association between tuberculosis and a hypercoagulable state, which may predispose patients to thrombotic complications such as deep vein thrombosis [38]. A study by Kutiyal et al. (2017) demonstrated that tuberculosis patients exhibit significant hemostasis abnormalities, including elevated D-dimer and fibrinogen levels, which indicate a hypercoagulable state that improves notably with anti-tubercular therapy [16]. Kager et al. (2017) demonstrated that pulmonary tuberculosis induces a systemic hypercoagulable state, characterized by increased levels of thrombin–antithrombin complexes, D-dimer, and fibrinogen, along with impaired anticoagulant mechanisms, highlighting the significant impact of tuberculosis on coagulation pathways [5].

Additionally, Factor VII levels are significantly lower, whereas Factor XII levels remain unchanged in TB patients [37].

The expression of tissue factor in macrophages is an important mechanism contributing to the hypercoagulable state in TB. TF plays a pivotal role in initiating the extrinsic coagulation pathway, resulting in thrombin generation and fibrin deposition. This increased pro-coagulant activity is particularly evident within granulomas, where TF expression is essential for granuloma formation and containment of *Mycobacterium tuberculosis*. Elevated levels of pro-inflammatory cytokines, such as tumor necrosis factor-alpha, interferon-gamma, interleukin-6, and interleukin-1 beta (IL-1β), contribute to the inflammatory response and upregulate TF expression in both macrophages and endothelial cells [19,36].

TB also alters the balance between pro-coagulant and anticoagulant factors. Patients with TB often exhibit increased levels of fibrinogen, factor VIII, and von Willebrand factor, all of which contribute to enhanced clot formation. Concurrently, decreased levels of natural anticoagulants such as protein C, protein S, and antithrombin III impair the body’s ability to regulate coagulation, further exacerbating the risk of thrombosis and thromboembolic events [36,39,40]. The C4b binding protein of the complement system is an acute-phase reactant that increases in concentration in inflammatory states [41]. Protein S in the bound form is complexed to C4b binding protein and is functionally inactive. As a result, the activity of free protein S is reduced in these conditions, enhancing the likelihood of thrombosis [42]. The combined effect of elevated pro-coagulant factors and reduced anticoagulant activity results in a hypercoagulable state that complicates TB management and increases morbidity and mortality.

Understanding these alterations is essential for managing thrombotic risks in TB patients. Effective TB treatment, along with appropriate anticoagulation and anti-inflammatory strategies, can help mitigate these risks. Targeting the underlying inflammation and balancing pro-coagulant and anticoagulant factors are essential components of managing the hypercoagulable state associated with TB [19,37].

### 2.5. Suppressed Fibrinolysis

Suppressed fibrinolysis in tuberculosis is a significant factor that contributes to the hypercoagulable state and increased risk of thrombotic complications. Tuberculosis is well known for its complex interactions with the immune and coagulation systems, resulting in a prothrombotic state and impaired fibrinolysis [36]. The hypercoagulable state in TB is characterized by elevated plasma fibrinogen levels, increased fibrinogen degradation products (FDPs), higher concentrations of tissue plasminogen activator (t-PA), and elevated levels of plasminogen activator inhibitor-1 (PAI-1). These factors collectively impair fibrinolysis and promote clot formation, significantly increasing the risk of thrombotic events such as deep venous thrombosis and pulmonary embolism [43].

The hypercoagulable state is accompanied by homeostatic disorders that lead to the suppression of fibrinolysis in TB patients. Studies have shown that extensive pulmonary TB is associated with significant suppression of the fibrinolytic system and reduced immunologic reactivity. Suppressed fibrinolysis is therefore a critical factor in the hypercoagulable state and contributes to disease severity and complications [17,44].

The interconnection between immune activation and fibrinolysis is essential for understanding the hypercoagulable state in TB. In patients with disseminated TB, a marked decrease in plasminogen activator activity is observed, accompanied by elevated fibrin degradation products, indicating impaired fibrinolytic function. The relationship between immune reactivity and fibrinolysis is highlighted by altered lymphocyte responses, including decreased blast transformation in response to phytohemagglutinin (PHA) and increased transformation when exposed to purified protein derivative (PPD). These findings suggest a complex interplay between immune activation and fibrinolytic suppression in TB patients [45].

Plasminogen activator inhibitor type-1 is a key regulator of fibrinolysis and inflammation in TB patients. Elevated plasma levels of PAI-1, consistently observed in TB, play a pivotal role in TB-associated coagulopathy by impairing fibrinolysis. By inhibiting t-PA, PAI-1 impairs the conversion of plasminogen to plasmin, reducing fibrin degradation and promoting persistent clot formation. Murine models of TB have shown that PAI-1 deficiency results in altered immune responses and higher bacterial loads in the lungs, further highlighting the role of PAI-1 not only in coagulation but also in modulating immune responses during the early stages of TB infection [10,43].

Suppressed fibrinolysis in TB is a multifactorial issue involving hypercoagulable states, homeostatic imbalances, and intricate interactions between the immune and coagulation systems. Addressing these disruptions through targeted treatment approaches could potentially reduce the morbidity and mortality associated with thrombotic complications in TB patients [17,43]. Further research is necessary to fully elucidate these mechanisms and develop effective treatments to counteract the hypercoagulable state and suppressed fibrinolysis observed in TB. Understanding these pathways may pave the way for more effective interventions to mitigate the thrombotic risks associated with TB.

### 2.6. Interaction with Immune Cells

The interaction between *Mycobacterium tuberculosis* and immune cells, particularly monocytes and macrophages, plays a critical role in the immune response to tuberculosis. This interaction is vital for understanding the pathogenesis of TB as well as the mechanisms of immune evasion that allow *Mycobacterium tuberculosis* to persist within the host [46]. Upon infection, macrophages and dendritic cells (DCs) internalize *Mycobacterium tuberculosis*, leading to activation and maturation. Macrophages secrete pro-inflammatory cytokines such as TNF-α, IL-1, and IL-6, while DCs produce IL-12 and IFN-α, which are essential for inducing IFN-γ production by T cells [47]. Additionally, macrophages produce IL-10, an immunosuppressive cytokine that inhibits IL-12 synthesis. Neutralizing IL-10 in Mtb-infected macrophages restores IL-12 secretion, highlighting the regulatory role of IL-10 in modulating immune responses [48,49].

*Mycobacterium tuberculosis* can manipulate phagolysosome fusion within macrophages, which allows it to evade the host’s microbicidal mechanisms. Virulent strains such as H37Rv are capable of escaping from fused phagolysosomes into nonfused vesicles or even the cytoplasm, thereby allowing intracellular replication. Moreover, *Mycobacterium tuberculosis* infection induces a biphasic metabolic response in macrophages. Initially, infected macrophages switch to aerobic glycolysis (the Warburg effect) and upregulate oxidative defense pathways. Later, these cells transition back to mitochondrial oxidative metabolism, which reduces pro-inflammatory responses and facilitates Mtb persistence. The ability to induce apoptosis is another immune evasion mechanism employed by Mtb [49,50]. The bacterium can trigger apoptosis in macrophages, particularly in alveolar macrophages from patients with TB and AIDS, a process that is specific to virulent strains like H37Rv and requires viable bacteria. Additionally, Mtb cell-wall glycolipids, such as mannose-capped lipoarabinomannan (ManLAM), inhibit CD4+ T cell activation by disrupting the immunological synapse between T cells and macrophages [51].

Macrophage responses to *Mycobacterium tuberculosis* also vary based on cell origin. Alveolar macrophages (AMs) and monocyte-derived macrophages (MDMs) exhibit different responses to infection. AMs tend to provide a niche for Mtb replication even after the onset of adaptive immunity, whereas MDMs display enhanced antibacterial activity and pro-inflammatory responses [52]. Granuloma formation is a hallmark of the immune response to TB. Granulomas are organized structures composed of various immune cells, including macrophages, and play a crucial role in containing and isolating *Mycobacterium tuberculosis*. However, granuloma formation can lead to localized tissue hypoxia due to restricted blood flow and vascular changes [53,54]. Hypoxia and subsequent endothelial injury within granulomas exacerbate the prothrombotic environment by damaging endothelial cells and impairing their anticoagulant properties. These effects collectively promote thrombosis, adding to the hypercoagulable state commonly observed in TB patients.

In addition to their role in immune activation, activated monocytes and macrophages also contribute to coagulation. These cells produce tissue factor, which initiates the extrinsic coagulation pathway, promoting thrombin generation and fibrin clot formation [19]. The production of TF serves as a link between inflammation and coagulation, increasing the risk of thrombosis. The complex interplay between immune activation, tissue injury, and increased thrombotic risk is a critical aspect of TB pathogenesis and progression. While granulomas are essential for containing the infection, they also contribute to localized tissue injury and the hypercoagulable state [10,55].

In conclusion, the interaction between *Mycobacterium tuberculosis* and immune cells involves intricate mechanisms of activation, cytokine production, phagolysosome manipulation, metabolic adaptation, and immune evasion. These interactions are also closely linked to coagulation pathways, contributing to thrombotic complications in TB patients. Understanding these processes is essential for developing effective treatments and vaccines for TB, as they highlight the dual roles of immune activation and evasion in the disease’s pathogenesis [52].

### 2.7. Hypoxia-Induced Changes

Hypoxia, especially prevalent in advanced pulmonary tuberculosis (TB), plays a significant role in exacerbating the hypercoagulable state associated with the disease. As the infection progresses, the formation of granulomas and the resulting lung tissue damage reduce oxygen supply, creating hypoxic conditions in the affected areas. Hypoxia stimulates the production of hypoxia-inducible factor-1 (HIF-1), a transcription factor that orchestrates the cellular response to low oxygen levels [56].

HIF-1 upregulates the expression of several pro-coagulant factors, contributing to an imbalance between coagulation and anticoagulation. One of the key effects of HIF-1 is the increased production of tissue factor, a primary initiator of the extrinsic coagulation pathway, which leads to enhanced thrombin generation and fibrin formation, ultimately promoting clot formation [57]. Additionally, HIF-1 can stimulate the production of other pro-coagulant proteins, such as plasminogen activator inhibitor-1, which further contributes to impaired fibrinolysis—the process responsible for breaking down clots.

The inhibition of fibrinolysis by PAI-1 is particularly concerning because it prevents the effective breakdown of fibrin clots, leading to their persistence and growth and increasing the risk of thrombus formation. The combination of increased pro-coagulant activity and reduced fibrinolytic capacity under hypoxic conditions creates a favorable environment for thrombus development. This is especially problematic in TB patients, where already existing inflammation and endothelial injury compound these risks [58].

Overall, hypoxia-induced HIF-1 activation plays a vital role in promoting hypercoagulability in TB by simultaneously enhancing pro-coagulant factor production and inhibiting the fibrinolytic pathway. This contributes to an elevated risk of thromboembolic complications, particularly in individuals with advanced pulmonary TB, who are more likely to experience extensive lung damage and associated hypoxic conditions. The interplay between hypoxia, inflammation, and coagulation represents a key mechanism underlying the prothrombotic state seen in TB, complicating both the course of the disease and its management [59].

## 3. Clinical Risks Associated with Hypercoagulability in TB

**Figure 2:**
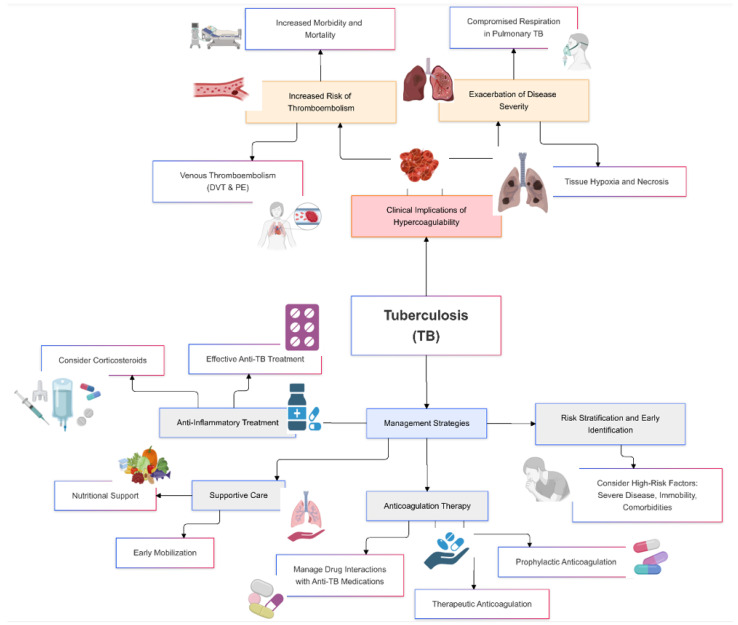
Clinical implications and management of hypercoagulability in tuberculosis.

Hypercoagulability in tuberculosis poses significant clinical risks, primarily due to the increased likelihood of thrombotic events such as venous thromboembolism, deep vein thrombosis, and pulmonary embolism [53]. These complications can exacerbate the severity of TB, lead to prolonged hospitalization, and increase morbidity and mortality rates (Figure 2).

### 3.1. Increased Risk of Thromboembolism

The hypercoagulable state in tuberculosis patients substantially increases the risk of venous thromboembolism, driven by factors such as systemic inflammation, endothelial dysfunction, enhanced platelet activation, and an imbalance between pro-coagulant and anticoagulant mechanisms [53]. Elevated levels of inflammatory cytokines, such as interleukin-6, stimulate the coagulation cascade and promote clot formation, which can lead to venous thrombosis [10,60].

Deep vein thrombosis occurs when a blood clot forms in the deep veins, often in the legs, and poses a significant threat to TB patients, particularly those who are immobilized or bedridden due to advanced disease [55]. The increased production of fibrinogen and other coagulation factors, along with reduced fibrinolytic activity, promotes clot formation in these deep veins. If a portion of a thrombus dislodges, it can travel to the lungs and cause a pulmonary embolism, which is a life-threatening condition that blocks blood flow in the pulmonary arteries [60].

Thromboembolic complications such as deep vein thrombosis (DVT) and pulmonary embolism can significantly worsen the prognosis of tuberculosis, leading to increased morbidity and mortality. This risk is particularly pronounced in patients with advanced or disseminated TB, where extensive tissue involvement, hypoxia, and widespread inflammation further elevate the risk of thromboembolism [36,60]. The presence of venous thromboembolism in TB patients also complicates clinical management, as anticoagulation therapy may be required. This presents challenges due to potential interactions with TB medications or an increased risk of bleeding, highlighting the need for early recognition and preventive measures to manage thromboembolic risks effectively.

The hypercoagulable state and suppression of fibrinolysis in TB patients carry significant clinical implications. The combination of elevated fibrinogen, increased levels of plasminogen activator inhibitor-1, and reduced natural anticoagulants such as protein C, protein S, and antithrombin III creates an environment that favors clot formation [37]. This increases the risk of thromboembolic events such as DVT and PE, complicating TB treatment, especially in patients with severe or disseminated disease. A thorough understanding of these alterations is essential for managing thrombotic risks in TB patients, emphasizing the importance of targeted anticoagulant and anti-inflammatory therapies [35].

Effective management of the hypercoagulable state and suppressed fibrinolysis in TB requires a comprehensive approach. Addressing underlying inflammation through appropriate anti-TB therapy is essential for reducing cytokine levels and improving the balance between pro-coagulant and anticoagulant factors. Adjunctive anticoagulation therapy may be considered for patients at high risk of thromboembolic events, though this approach must be balanced against the risk of bleeding, particularly when anticoagulants interact with TB medications. Further research is needed to develop therapeutic interventions that specifically target elevated PAI-1 levels and enhance fibrinolytic activity in TB patients, ultimately mitigating the risks associated with the hypercoagulable state and improving clinical outcomes [53].

### 3.2. Exacerbation of Disease Severity

The hypercoagulable state in TB can also exacerbate disease severity by impairing blood flow to affected tissues, ultimately leading to tissue hypoxia and necrosis. The formation of thrombi within blood vessels, particularly those supplying areas involved in granulomatous inflammation, can further restrict oxygen delivery to already compromised tissues. This reduced blood flow results in localized tissue hypoxia, which impairs the ability of immune cells to effectively control the infection and can contribute to tissue damage and necrosis.

In pulmonary TB, thrombosis of pulmonary vessels can further compromise respiratory function. The formation of clots in the pulmonary vasculature restricts blood flow through the lungs, reducing oxygen exchange and leading to worsening hypoxia. This can be particularly detrimental in patients with extensive lung involvement, where impaired blood flow and oxygenation can significantly impact clinical outcomes. The resulting hypoxia can also stimulate the production of hypoxia-inducible factor-1, which further promotes a pro-coagulant state, creating a vicious cycle that exacerbates both coagulation and inflammation.

In addition, impaired blood flow due to thrombosis may hinder the delivery of therapeutic agents to infected tissues, reducing the efficacy of TB treatment. This can contribute to prolonged disease progression and an increased risk of complications. The combined effects of hypercoagulability, tissue hypoxia, and impaired immune function create a challenging environment for effective TB management, particularly in patients with advanced disease. Addressing the hypercoagulable state through targeted interventions, such as anticoagulant therapy, may help improve clinical outcomes and reduce the severity of TB-related complications.

## 4. Clinical Implications and Management Strategies

### 4.1. Risk Stratification and Early Identification

Identifying tuberculosis patients at high risk for thromboembolic events (VTEs) is essential for preventing complications and improving outcomes. The increased risk of VTE in TB patients is driven by multiple factors, including systemic inflammation, immobility, comorbidities, and endothelial dysfunction. Therefore, effective risk stratification is very important in guiding the management of these patients to mitigate thrombotic complications [61]. Current research highlights the need for specialized risk assessment models (RAMs) tailored to TB patients, as general models may not sufficiently capture the unique risk factors associated with TB [62].

General risk assessment models, such as the Padua and Geneva RAMs, have been commonly used in the general population to predict the risk of VTE [61]. However, these models show minimal positive predictive value for TB patients. In a study involving 865 smear-positive TB patients, 37 developed VTE. The Padua model predicted 29.7% of cases, while the Geneva model predicted 32.4%, but neither considered TB as an independent risk factor. This limitation underscores the need for TB-specific RAMs that can accurately assess thrombotic risk in this unique patient population [61].

Recent advancements in personalized risk predictors show promise in addressing the gaps left by general RAMs. The PERISKOPE-TB personalized risk predictor, which combines T-cell sensitization measures with clinical covariates, estimates the risk of developing active TB in individuals with latent TB infection (LTBI) [63]. PERISKOPE-TB is a personalized risk prediction tool developed through a large international collaboration, led by researchers at University College London. The project was funded by the National Institute for Health Research (NIHR). It demonstrated a high predictive accuracy with a C-statistic of 0.88, highlighting its utility in targeting preventative treatment. Additionally, a three-gene transcriptional signature (BATF2, GBP5, SCARF1) was identified to predict the short-term risk of active TB with a positive predictive value (PPV) of 50% and a negative predictive value (NPV) of 99.3% [64]. This blood transcriptional biomarker outperformed traditional interferon-gamma release assays, offering an improved approach for identifying individuals at higher risk of progressing to active TB.

Risk stratification tools have also been developed to tailor treatment approaches to individual TB patients. A six-item risk score, developed from phase 3 clinical trial data, uses parameters such as HIV status, smear grade, sex, cavitary disease status, body mass index, and Month 2 culture status to classify TB patients into low-, moderate-, and high-risk groups [62]. This stratification helps determine the optimal treatment duration, with high-risk groups typically requiring extended therapy. Screening and diagnostic approaches have also been refined to improve risk identification. A multiplexed cytokine biosensor assay that profiles cytokine release to identify biomarker signatures for LTBI status and reactivation risk achieved predictive accuracies of over 80%, highlighting its potential to enhance risk stratification for TB patients [65]. Furthermore, combining the tuberculin skin test (TST) and T.Spot with risk stratification tables improved the identification of high-risk patients, particularly those on immunosuppressants, ensuring that appropriate preventative treatments such as chemoprophylaxis are provided to those who need them most [66].

Effective risk stratification for thromboembolism in TB patients involves evaluating specific patient characteristics and recognizing key risk factors that increase the likelihood of thrombotic complications. Severe TB disease, particularly with extensive pulmonary involvement or dissemination to other organs, is associated with a heightened risk of hypercoagulability due to the intense systemic inflammatory response. Immobility, whether from hospitalization, bed rest, or physical limitations, also significantly contributes to thrombotic risk by promoting stasis of blood flow, particularly in the lower extremities. Comorbidities such as HIV infection and diabetes further elevate the risk of VTE in TB patients [64]. HIV-related immune dysregulation exacerbates inflammation, while diabetes is associated with endothelial dysfunction and impaired blood flow. Additional factors that increase thrombotic risk include a history of thrombosis, obesity, or the use of certain medications that influence coagulation.

The identification of TB patients at high risk for VTE is critical for implementing timely and effective preventive measures, such as anticoagulation therapy or supportive care. Anticoagulation therapy, while potentially beneficial, must be carefully managed in the context of TB treatment, as interactions with anti-TB medications can increase the risk of bleeding or reduce the efficacy of therapy. Supportive care measures, such as early mobilization, management of comorbidities, and nutritional support, are also essential components of reducing thrombotic risk in TB patients [63,64].

Current general RAMs such as the Padua and Geneva models are insufficient for predicting VTE in TB patients, necessitating the development of TB-specific risk models. Personalized risk predictors and advanced diagnostic tools, including PERISKOPE-TB and multiplexed cytokine biosensor assays, show promise in improving risk stratification and targeting preventative treatments effectively [63]. The combination of screening methods, such as TST and T.Spot, and the implementation of TB-specific risk stratification tools may enhance the identification of high-risk patients, ensuring timely and appropriate interventions. Understanding these risk factors and using tailored approaches to manage thrombotic risks in TB patients is essential for improving clinical outcomes and reducing complications associated with the disease [65].

### 4.2. Anticoagulation Therapy

**Figure 3:**
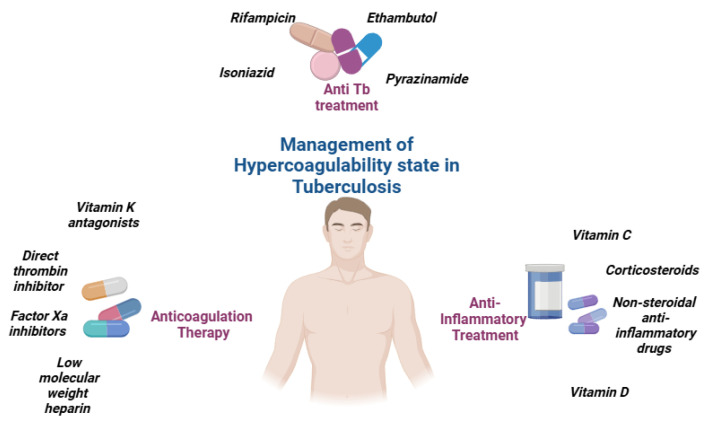
Management of hypercoagulability state in tuberculosis.

Anticoagulation therapy is a critical component of care for tuberculosis patients who experience venous thromboembolic events (VTEs). However, managing anticoagulation in TB patients presents unique challenges, particularly due to drug interactions between traditional anticoagulants, such as Vitamin K antagonists (VKAs), and anti-TB medications like rifampicin (Figure 3).

The use of VKAs, such as warfarin, in TB patients is complicated by significant drug–drug interactions, particularly with rifampicin, a cornerstone of TB treatment. Rifampicin induces liver enzymes responsible for the metabolism of VKAs, reducing their anticoagulant effect and necessitating frequent dose adjustments [67]. As a result, patients on VKAs require regular monitoring of the International Normalized Ratio (INR) to maintain a therapeutic range, which can be burdensome and difficult to achieve consistently. This complexity makes the management of anticoagulation challenging in TB patients, particularly those with additional contraindications or bleeding risks.

Novel oral anticoagulants (NOAs) such as a direct thrombin inhibitor (dabigatran) and factor Xa inhibitors (rivaroxaban, apixaban, and edoxaban) present a promising alternative to VKAs for TB patients with VTE. NOAs, which include direct oral anticoagulants (DOACs), do not require frequent laboratory monitoring, dosing adjustments, or dietary restrictions, and they have fewer drug interactions compared to VKAs. These features make NOAs an attractive option for TB patients requiring anticoagulation therapy. However, despite their theoretical benefits, there is currently a lack of published data assessing the safety and efficacy of NOAs in combination with anti-TB antimicrobials, including rifampicin. This gap in research highlights the need for further studies to establish the role of NOAs in managing thromboembolic events in TB patients [67,68].

**Figure 4:**
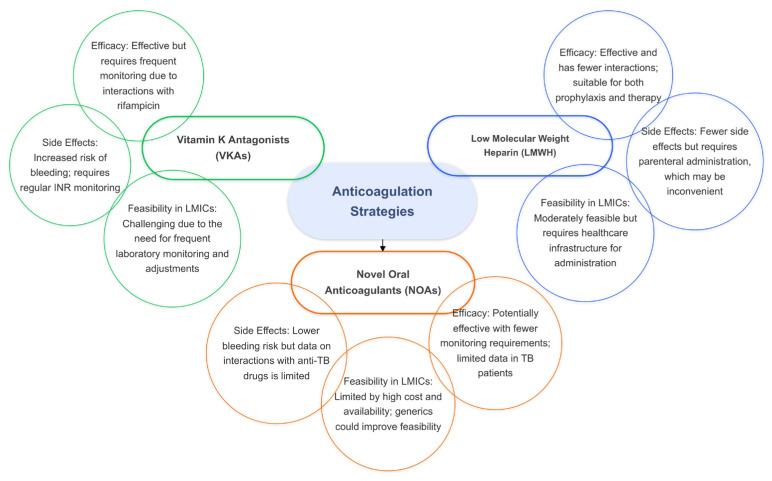
The efficacy, side effects, and feasibility of anticoagulation strategies in tuberculosis.

In the absence of sufficient evidence for NOAs, low-molecular-weight heparin (LMWH) may serve as a viable alternative to VKAs for preventing VTE complications in TB patients. LMWH has the advantage of fewer drug interactions compared to VKAs and can be used as both a prophylactic and therapeutic anticoagulant. However, the parenteral route of administration for LMWH can be inconvenient for some patients, especially for long-term use. Despite this limitation, LMWH remains a potential option for TB patients who require anticoagulation but cannot tolerate VKAs or for whom NOAs are not suitable (Figure 4).

For TB patients at high risk of thrombosis, prophylactic anticoagulation may be considered to prevent thromboembolic events. Hospitalized or immobilized TB patients are particularly vulnerable to venous stasis and clot formation, making prophylactic anticoagulation important for this population. LMWH or other anticoagulants may be used as preventive measures to decrease the risk of deep vein thrombosis and pulmonary embolism. Careful monitoring and individualized assessment are essential to ensure that the benefits of prophylactic anticoagulation outweigh the risks, particularly in TB patients with bleeding tendencies or other contraindications [67,68].

For patients with confirmed thromboembolic events, therapeutic anticoagulation is recommended. However, the management of anticoagulation in TB patients is complex due to potential interactions with anti-TB medications, particularly rifampicin, which reduces the efficacy of anticoagulants and necessitates close monitoring. Frequent assessments of coagulation status and collaboration between healthcare providers are essential to safely manage anticoagulation therapy in TB patients, balancing the need to prevent clot-related complications with the risk of bleeding [68].

While NOAs present a promising alternative to VKAs for TB patients with VTE, the lack of robust clinical data necessitates further research to establish their safety and efficacy. LMWH remains a potential option despite challenges related to its administration. Effective management of anticoagulation therapy in TB patients requires careful consideration of drug interactions, close monitoring of anticoagulation status, and individualized risk assessments. Addressing these gaps in research and optimizing anticoagulation strategies are essential for improving outcomes in TB patients with thromboembolic complications [67].

### 4.3. Anti-Inflammatory Treatment

Addressing systemic inflammation in tuberculosis patients is essential for mitigating the hypercoagulable state that increases the risk of thrombotic complications such as deep vein thrombosis and pulmonary embolism. Combining effective anti-tuberculosis (anti-TB) treatment with adjunctive anti-inflammatory therapies can significantly reduce systemic inflammation and lower the risk of clot formation, thereby improving clinical outcomes.

Vitamin D and vitamin C have emerged as promising adjunctive treatments in tuberculosis due to their immunomodulatory properties [69]. Studies have shown that supplementation with vitamin D and vitamin C significantly improves sputum smear conversion rates and accelerates radiographic resolution in TB patients, contributing to better clinical outcomes [70]. It also increases the lymphocyte-to-monocyte ratio, indicating a reduction in systemic inflammation. Host-directed therapies (HDTs) such as prednisolone and pentoxifylline have also demonstrated trends towards reducing levels of tumor necrosis factor-alpha, a key pro-inflammatory cytokine involved in TB pathogenesis [71,72]. These HDTs are generally well tolerated and effective in improving TB outcomes by modulating the immune response and controlling excessive inflammation.

Non-steroidal anti-inflammatory drugs (NSAIDs) have shown beneficial effects as adjunctive therapy in TB by reducing lung pathology and balancing the host immune response. This immunomodulatory effect can potentially reduce disease severity and improve treatment outcomes. NSAIDs may also inhibit actively replicating, dormant, and drug-resistant *Mycobacterium tuberculosis* cells, although the exact mechanisms behind their antimicrobial activity are still under investigation. Despite this, the use of NSAIDs as adjunctive therapy appears to provide significant clinical benefit by reducing excessive inflammation and lung tissue damage [73].

Corticosteroids are another important anti-inflammatory option, particularly for patients with severe manifestations of TB, such as TB meningitis or pericarditis. The use of corticosteroids can help control the inflammatory response, reduce tissue damage, and lower the risk of complications associated with hyperinflammation, including thrombosis. However, corticosteroids must be used with caution, as they carry potential side effects, including an increased risk of thrombotic events, hyperglycemia, and immunosuppression. Therefore, the decision to use corticosteroids must be individualized based on the patient’s clinical condition, with careful consideration of the potential benefits versus risks [74].

Platelets play a significant role in TB immunopathology, and their activation contributes to the hypercoagulable state commonly observed in TB patients. TB patients often exhibit high platelet counts, which correlate with disease severity. Platelets interact with immune cells and promote the secretion of matrix metalloproteinases (MMPs), contributing to tissue damage. Targeting platelet activation with anti-platelet agents could potentially limit tissue damage and reduce systemic inflammation in TB patients, thus lowering the hypercoagulable state and improving overall treatment outcomes.

The timing and balance of pro-inflammatory and anti-inflammatory responses are essential determinants of the immune outcome in TB. An excessive pro-inflammatory response can lead to tissue damage and exacerbate disease severity, while a balanced immune response helps contain the infection and prevent progression to active disease. Prompt initiation of anti-TB drugs, including isoniazid, rifampicin, pyrazinamide, and ethambutol, targets the underlying infection and subsequently reduces systemic inflammation by decreasing the release of pro-inflammatory cytokines such as IL-6. By effectively managing inflammation, these anti-TB drugs help to reduce the risk of clot formation and improve patient outcomes.

Combining anti-tuberculosis treatment with adjunctive anti-inflammatory therapies such as vitamin D, NSAIDs, corticosteroids, and anti-platelet agents can effectively reduce systemic inflammation, mitigate the hypercoagulable state, and improve outcomes in TB patients [73]. Anti-inflammatory treatments help balance the immune response, reduce tissue damage, and lower the risk of thrombotic complications, which are common in TB patients due to persistent systemic inflammation. The use of vitamin D and other host-directed therapies can enhance sputum conversion and radiographic resolution, while NSAIDs and corticosteroids can modulate immune responses to prevent excessive inflammation. Anti-platelet agents offer a targeted approach to reducing platelet-driven tissue damage and hypercoagulability. A comprehensive approach that addresses both infection control and inflammation management is essential for optimizing treatment outcomes and reducing the risk of complications in TB patients [75].

Further research is warranted to explore the full potential of these anti-inflammatory treatments, including their impact on thrombotic risk reduction in TB patients. Such studies will help to establish evidence-based strategies for incorporating these therapies into TB treatment regimens, thereby improving both disease outcomes and the quality of life for TB patients worldwide [68,73,74].

### 4.4. Supportive Care

Supportive care is an important component of managing hypercoagulability in TB patients, as it addresses underlying factors that contribute to thrombosis risk and enhances overall patient outcomes. Nutritional support is particularly important, as malnutrition is common among TB patients and can impair immune function and increase susceptibility to complications, including thromboembolism. A balanced diet rich in essential nutrients, vitamins, and minerals can help improve immune response, reduce inflammation, and support vascular health [76].

Early mobilization is another key aspect of supportive care in TB patients, especially those who are hospitalized or have limited physical activity. Prolonged immobility is a major risk factor for venous thromboembolism, as it leads to blood stasis and an increased risk of clot formation. Encouraging patients to engage in physical activity, even light movement or exercises, can help improve circulation, reduce venous stasis, and decrease thrombotic risk. In cases where patients are unable to mobilize independently, physical therapy or assisted movement exercises can be beneficial [5,77].

In addition to nutrition and mobilization, other supportive measures, such as ensuring adequate hydration and addressing comorbidities like diabetes or hypertension, are essential for reducing thrombotic risk. Comprehensive supportive care helps not only to mitigate hypercoagulability but also to enhance the overall recovery and quality of life for TB patients, ultimately contributing to better clinical outcomes and reduced complications [76].

### 4.5. Hypercoagulability Management Strategies in Resource-Limited Settings

TB disproportionately affects low- and middle-income countries, where healthcare systems are often under-resourced and constrained. This disparity creates significant challenges in implementing comprehensive management strategies for hypercoagulability in TB patients. In such settings, access to diagnostic tools for identifying thrombotic risks is limited, with minimal availability of laboratory tests like D-dimer, activated partial thromboplastin time (aPTT), and prothrombin time (PT). This limitation complicates the early identification of at-risk patients, emphasizing the need for simplified, low-cost diagnostic protocols that can be feasibly integrated into primary healthcare systems.

Anticoagulation therapy, while effective in managing hypercoagulability, presents unique obstacles in resource-constrained environments. Traditional therapies such as Vitamin K antagonists (VKAs) like warfarin require frequent monitoring and dose adjustments, which are challenging to achieve in settings with inadequate access to laboratory facilities. Additionally, interactions between VKAs and rifampicin, a cornerstone of TB treatment, necessitate more intensive follow-up. Novel oral anticoagulants (NOAs) offer a promising alternative due to their simplified dosing and reduced need for monitoring; however, their high cost and limited availability in low- and middle-income countries remain significant barriers. Exploring the potential for locally produced generics or subsidized programs for NOAs could enhance their accessibility and affordability.

Adjunctive therapies such as anti-inflammatory agents, including corticosteroids and NSAIDs, also require careful consideration in low- and middle-income countries. While these therapies can mitigate systemic inflammation and reduce thrombotic risk, they must be used judiciously to avoid side effects such as gastrointestinal bleeding and immune suppression, which are more concerning in resource-limited settings with poor access to secondary care. Vitamin D supplementation, a cost-effective adjunctive therapy, holds particular promise due to its immunomodulatory effects and ease of administration. Scaling up community-based initiatives for micronutrient supplementation could enhance outcomes without overburdening healthcare infrastructure.

Preventive strategies such as early mobilization and nutritional support offer low-cost, high-impact solutions for mitigating hypercoagulability in TB patients in low- and middle-income countries. Encouraging physical activity even within constrained environments can reduce venous stasis and thrombotic risk. Nutritional interventions, including community distribution of nutrient-dense foods, can address malnutrition, which exacerbates the hypercoagulable state in TB. Integrating these strategies into existing TB control programs, supported by local governments and international organizations, can enhance the feasibility and effectiveness of hypercoagulability management in resource-limited settings, ultimately improving patient outcomes.

## 5. Conclusions

The hypercoagulable state induced by tuberculosis is driven by a complex interplay between systemic inflammation, endothelial dysfunction, platelet activation, and impaired coagulation regulation. Addressing these factors through comprehensive management strategies including effective anti-TB therapy, targeted anticoagulation, and adjunctive anti-inflammatory treatments is essential for mitigating thrombotic risks and improving patient outcomes. Further research into the use of novel oral anticoagulants and host-directed therapies will help optimize anticoagulation approaches in TB patients. A tailored and multifaceted approach is necessary to reduce thromboembolic complications, ultimately improving both clinical outcomes and quality of life for individuals affected by TB.

## Data Availability

The data presented in this study are available upon request from the corresponding author.
